# High-latitude warming initiated the onset of the last deglaciation in the tropics

**DOI:** 10.1126/sciadv.aaw2610

**Published:** 2019-12-11

**Authors:** Margaret S. Jackson, Meredith A. Kelly, James M. Russell, Alice M. Doughty, Jennifer A. Howley, Jonathan W. Chipman, David Cavagnaro, Bob Nakileza, Susan R. H. Zimmerman

**Affiliations:** 1 Department of Earth Sciences, Dartmouth College, Hanover, NH 03755, USA.; 2 Department of Earth, Environmental, and Planetary Sciences, Brown University, Providence, RI 02912, USA.; 3 Geology Department, Bates College, Lewiston, ME 04240, USA.; 4 Mountain Resource Centre, Makerere University, Kampala, Uganda.; 5 Center for Accelerator Mass Spectrometry, Lawrence Livermore National Laboratory, Livermore, CA 94550, USA.

## Abstract

Atmospheric greenhouse gas concentrations are thought to have synchronized global temperatures during Pleistocene glacial–interglacial cycles, yet their impact relative to changes in high-latitude insolation and ice-sheet extent remains poorly constrained. Here, we use tropical glacial fluctuations to assess the timing of low-latitude temperature changes relative to global climate forcings. We report ^10^Be ages of moraines in tropical East Africa and South America and show that glaciers reached their maxima at ~29 to 20 ka, during the global Last Glacial Maximum. Tropical glacial recession was underway by 20 ka, before the rapid CO_2_ rise at ~18.2 ka. This “early” tropical warming was influenced by rising high-latitude insolation and coincident ice-sheet recession in both polar regions, which lowered the meridional thermal gradient and reduced tropical heat export to the high latitudes.

## Teaser

Tropical glaciers retreated before deglacial greenhouse gas rise due to a lowered meridional thermal gradient.

## INTRODUCTION

The interhemispheric synchrony of the ice age cycles is one of the greatest questions in paleoclimate research. During much of the Pleistocene, Northern Hemisphere (NH) high-latitude summer insolation appears to have paced global glaciation, yet the mechanisms by which this climate response was propagated to the Southern Hemisphere (SH) are uncertain (*1*, *2*). The rapid terminations of ice ages complicate the problem, as these occurred during periods of both high- and low-amplitude NH high-latitude summer insolation changes (*3*).

Atmospheric greenhouse gases (GHGs), particularly CO_2_, are commonly invoked to explain the global synchrony of glacial-interglacial cycles (*4*). This hypothesis is supported by near-concurrent changes in atmospheric CO_2_ levels and Antarctic temperatures over the last ~800,000 years (*5*), and numerous studies point to atmospheric GHGs as a key factor in warming Earth during the last deglaciation (*6*–*8*). However, other climate processes may have also played a role in unifying global temperatures. For example, low NH high-latitude summer insolation and coincident high ice-sheet albedo altered the meridional thermal gradient, which likely affected the strength and position of wind belts (*9*). Such changes in atmospheric circulation may have altered the strength of atmospheric and oceanic heat transport during glacial periods and induced cooling separate from that caused by GHG changes (*9*). The interconnected nature of high-latitude and GHG forcings complicates efforts to assess the sensitivity of Earth’s climate system to changes in these boundary conditions.

The tropics are an ideal region in which to investigate the impact of global forcings on glacial-interglacial temperature change as they are far from the direct forcing from high-latitude insolation and large ice sheets (*10*, *11*). Tropical glaciers, in particular, provide a valuable record of past change in the low latitudes as these glaciers are highly sensitive to changes in temperature (see the Supplementary Materials) (*12*), and their past fluctuations reflect changes in mid-tropospheric temperature (*13*). Prior work using cosmogenic beryllium-10 (^10^Be) surface exposure dating and analyses of glacially influenced lake sediments indicates that some tropical glaciers achieved their maximum extents either before or early during the Last Glacial Maximum (LGM; ~26.5 to 19.0 ka) (*14*–*17*) and that deglaciation from their LGM maxima was underway by ~20 ka before the rapid CO_2_ rise at ~18.2 ka (*18*, *19*). Critically, these existing data are limited to tropical South America. It is therefore unknown whether these records reflect temperature across the wider tropics or more regional conditions. In addition, the recent determination of low-latitude, high-elevation cosmogenic nuclide production rates (*20*, *21*) requires recalculation of ^10^Be ages from tropical South America to allow for accurate comparison with global records.

**Fig. 1:**
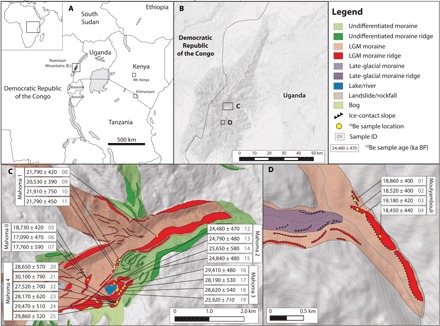
The Rwenzori Mountains and geomorphic maps of field areas. (**A**) The Rwenzori Mountains (boxed) occur on the border between Uganda and the Democratic Republic of the Congo. (**B**) They are an uplifted horst of basement rock in the western branch of the East African Rift System. We targeted two separate catchments for glacial chronology: (**C**) the lower Mubuku valley and (**D**) the Moulyambouli valley. Sample locations are yellow circles. ^10^Be ages are in years ago with internal, 1σ uncertainties. Numbers in gray are the map ID number of samples in tables S1 to S3. One outlier (sample 19) is shown in italics.

**Fig. 2:**
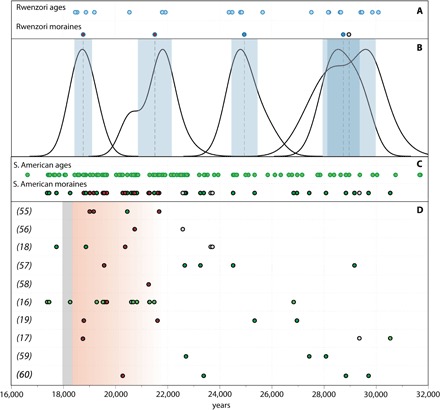
The Rwenzori (Mahoma 1 to 4 and Moulyambouli moraines) and recalculated tropical South American LGM moraine chronologies. (**A**) Light blue circles are individual Rwenzori ^10^Be ages. Dark blue circles are mean moraine ages. (**B**) Rwenzori moraine ages as normalized probability (camel) plots with 1σ, internal uncertainties highlighted in blue. (**C**) Recalculated tropical South American ^10^Be ages (see the Supplementary Materials for details; ID numbers in this figure correspond to assigned IDs in site descriptions in the Supplementary Materials). Light green circles are individual ^10^Be ages, and dark green circles are mean moraine ages. For both the Rwenzori and tropical South American moraine chronologies, gray circles mark the outermost moraine in a given catchment, if dated. Red circles mark the onset of recession from or from near the LGM maximum ice extent in each catchment. (**D**) Mean moraine ages grouped by original study. The red-shaded area highlights the onset of glacial recession in the Rwenzori and tropical South America. The gray bar indicates the timing of the CO_2_ rise at ~18.2 ka (*34*).

To assess the timing of glacial fluctuations across the tropics during the LGM, we determined a chronology of past glacial extents using ^10^Be dating of moraines in tropical East Africa, far from the South American tropics. Our ^10^Be chronology includes 17 new and 8 previously published ages (*22*) that constrain the timing of glacial fluctuations in the equatorial Rwenzori Mountains (~0.3°N, 30.0°E), located on the border between Uganda and the Democratic Republic of the Congo, during the LGM and the onset of deglaciation (Fig. 1). We also recalculated 177 ^10^Be ages of 48 LGM moraines in tropical South America from 10 prior studies (nine sites) (Fig. 2 and the Supplementary Materials). All ages are calculated using a low-latitude, high-altitude ^10^Be production rate (*21*) and time-independent (“St”) scaling (see Materials and Methods) (*23*–*25*). There are notable uncertainties in tropical cosmogenic nuclide production rate scaling during the LGM (*26*). Although these uncertainties affect comparisons of tropical ^10^Be glacial chronologies with other paleoclimate records, they do not affect the larger conclusions of this work. All ^10^Be ages reported here are of boulders on the crests of moraines, which we infer as representing the final sedimentation on a moraine. Therefore, we interpret a ^10^Be age as the time of glacial recession from a given moraine and, thus, the onset of warming.

## RESULTS

During the last ice age, glaciers from the central peaks in the Rwenzori Mountains flowed down the Bujuku and Mubuku valleys and merged to form a single glacier in the lower Mubuku valley that terminated at ~2000-m elevation (Fig. 1). At its maximum extent, the glacier overtopped the right-lateral (south) valley wall, flowed ~500 m southward, and formed a series of moraines that enclose Lake Mahoma at ~3000 meters above sea level (m.a.s.l.) (Fig. 1 and tables S1 to S3). We term these moraines, from the outermost to innermost, the Mahoma 4, 3, 2, and 1 moraines. ^10^Be ages of the two outermost moraines yield mean ages of 28,960 ± 1020 years ago (Mahoma 4; *n* = 6) and 28,740 ± 620 years ago (Mahoma 3; *n* = 3; one outlier omitted). Inboard of the Mahoma 3 moraine, the Mahoma 2 moraine has a mean age of 24,940 ± 500 years ago (*n* = 4) (*22*). The Mahoma 2 moraine is cross cut by the largest of these moraines, Mahoma 1, which has a mean age of 21,500 ± 650 years ago (*n* = 4) (*22*) and extends down the Mubuku valley to ~2300 m.a.s.l. The Mahoma 1 moraine has the greatest relief of any moraine within the series (~150 m relief above the Mubuku river valley floor) and marks the farthest down-valley extent of ice in the Mubuku valley during the last ice age. Approximately 5 km up the Mubuku valley from the Mahoma 1 terminus, the Mahoma 0 moraine has a mean age of 17,860 ± 830 years ago (*n* = 3).

We also dated a single large (30 to 50 m relief above the valley floor), sharp-crested moraine in the Moulyambouli valley, ~10 km south of the Mubuku valley (Fig. 1). While there are smaller, partially preserved moraine segments on the steep bedrock valley wall opposite the large Moulyambouli moraine, there is no series of moraines comparable to that in the lower Mubuku valley. ^10^Be ages of the Moulyambouli moraine yield a mean age of 18,750 ± 340 years ago (*n* = 4). Because there is only a single moraine dated in the Moulyambouli valley, we rely more heavily on the moraine chronology from the lower Mubuku valley where we can track glacial fluctuations throughout the LGM. Together, the Mahoma and Moulyambouli moraine chronologies indicate that Rwenzori glaciers reached their maximum extents by ~28 ka. The glaciers fluctuated near their maxima until ~21.5 ka in the Mubuku valley and ~18.8 ka in the Moulyambouli valley, at which point deglaciation was underway in both catchments.

A comparison of the Rwenzori moraine chronology with 48 recalculated moraine ages from nine sites in tropical South America shows a broad similarity in the timing and structure of tropical glacial fluctuations during the LGM (Fig. 2 and the Supplementary Materials). South American glaciers fluctuated throughout the LGM, with some glaciers achieving their maximum extents by ~28 to 29 ka. Similar to the Rwenzori glaciers, the tropical South American glaciers retreated from or from near their LGM maxima by ~20 to 19 ka. Together, the Rwenzori and South American moraine chronologies show a coherent signal of cool tropical temperatures during the LGM until the onset of glacial recession, indicating warming at ~20 to 19 ka.

## DISCUSSION

**Fig. 3:**
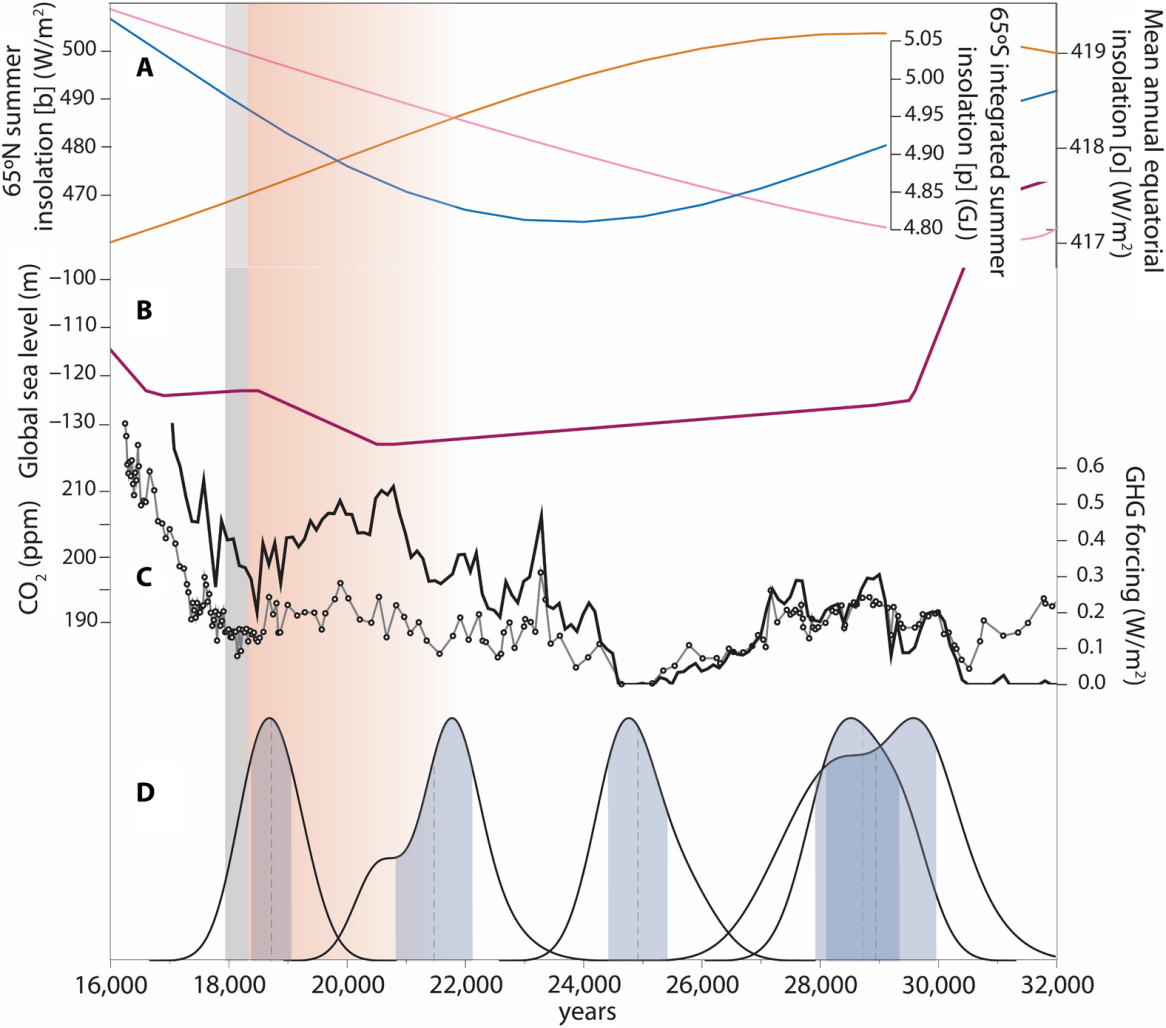
Global boundary conditions during the LGM and Rwenzori moraine ages. (**A**) NH high-latitude summer insolation (blue), mean annual equatorial insolation (orange), and integrated SH high-latitude summer insolation (pink) (*54*); (**B**) global sea level (*28*); and (**C**) normalized GHG forcing from CO_2_, methane, and N_2_O (*29*–*31*). Atmospheric CO_2_ concentration is shown independently (linked black circles). (**D**) The Rwenzori glacial chronology (as in Fig. 2) indicates expanded ice coincident with low values of each of the global boundary conditions (A to C). The red-shaded area and gray bar indicate the onset of tropical glacial recession and rapid atmospheric CO_2_ rise, respectively, as in Fig. 2.

The coincidence of tropical glacial maxima with a minimum in NH high-latitude summer insolation, high global ice-sheet volume, and low atmospheric GHG concentrations shows that tropical temperatures were sensitive to these global climate forcings during the LGM (Fig. 3). Milankovitch theory holds that low NH high-latitude summer insolation enabled the growth of NH ice sheets, which, in turn, cooled the planet via increased albedo (*8*, *27*). Because late Pleistocene sea-level changes largely reflect changes in NH ice sheets (*28*), we use sea level as a proxy for NH ice-sheet volume. By this metric, the period of most extensive NH ice sheets (~29 to 19 ka), and likely the period of greatest ice-sheet albedo forcing, is coincident with the time of expanded tropical glaciers. GHG radiative forcing was also low from ~34 to 18 ka (Fig. 3) (*29*–*31*), which presumably reinforced tropical cooling during the LGM (*32*), particularly at high altitudes (*33*).

**Fig. 4:**
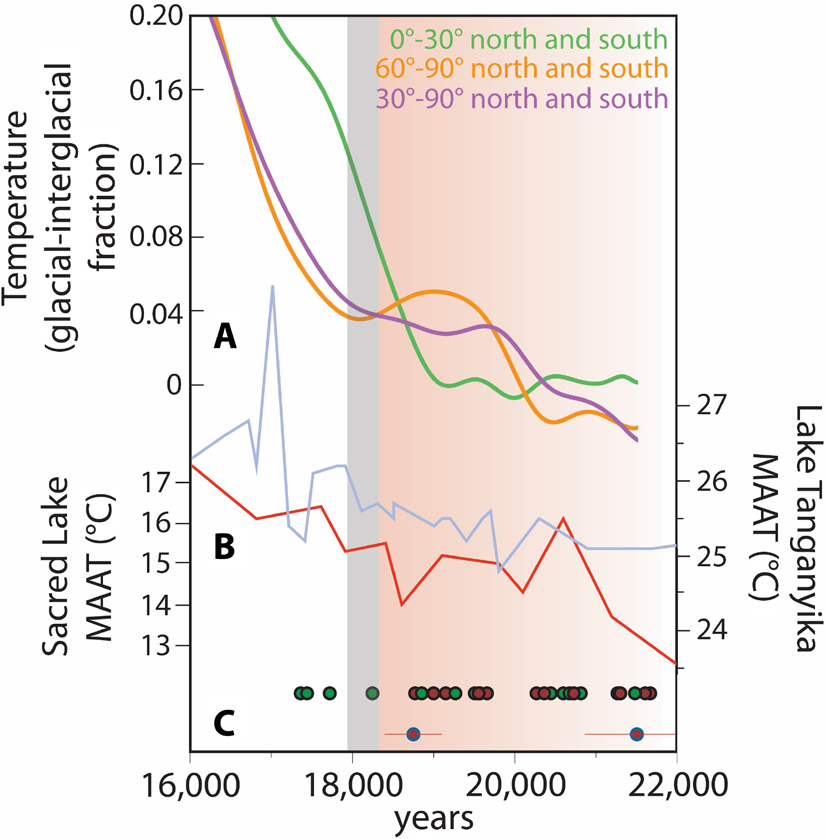
Longitudinal temperature changes at the onset of the last deglaciation from syntheses of global temperature records. (**A**) Average NH and SH temperature changes relative to changes in the low latitudes. All values plotted versus the net fraction of glacial-interglacial temperature change, as in (*7*). (**B**) Organic geochemical temperature reconstructions from tropical African lakes Tanganyika (light blue) (*35*) and Sacred (red) (*36*), as plotted by Loomis *et al.* (*33*). (**C**) Tropical moraine ages as in Fig. 2, with tropical South American mean moraine ages at the top and Rwenzori moraine ages (with 1σ error) below.

Although atmospheric GHG concentrations influenced tropical cooling during the LGM, the glacial chronologies presented here indicate that tropical warming began “early” (i.e., at ~20 to 19 ka) before the rapid rise of CO_2_ at ~18.2 ka (*34*) that is hypothesized to have influenced global deglaciation [e.g., (*3*)]. The early warming registered by tropical glaciers is supported by temperature reconstructions from East African lake sediments that document the onset of deglacial warming at ~20 ka (Fig. 4) (*35*, *36*). Moreover, synthesized tropical sea surface temperature (SST) records (*7*) indicate that warming was underway across the tropical ocean (30°N-30°S) by at least ~19 ka (Fig. 4). This early onset of tropical warming does not align with changes in atmospheric GHG levels or local insolation. GHG radiative forcing rose by ~0.5 W/m^2^ between ~25 and 20 ka but stagnated or fell between ~20 and 18 ka, a time when tropical glaciers receded and temperatures increased (Figs. 3 and 4). In addition, mean annual equatorial insolation decreased by ~2.0 W/m^2^ between ~30 and 16 ka. Although the rate and magnitude of change were low, decreasing insolation would have encouraged glacial advance throughout this period, including at ~20 to 19 ka. An alternative mechanism is required to explain the timing of tropical warming at the end of the LGM.

Coincident with, or possibly before, the tropical warming at ~20 to 19 ka, NH and SH high-latitude regions warmed. The Laurentide Ice Sheet began to retreat from or from near its maximum extents before ~20 ka (*37*, *38*), driven by rising NH high-latitude summer insolation after ~24 ka (*38*). Deglaciation on the Antarctic Peninsula (*39*) and the onset of warming in West Antarctica (*40*) are dated to ~20 ka and are attributed to increasing SH summer insolation duration (*41*). The rise in global sea level at ~21 to 19 ka (*28*) likely reflects the recession of the large polar ice sheets. Compilations of other NH high- and mid-latitude terrestrial and oceanic records likewise suggest that warming was underway by ~20 ka; SH mid-latitude oceanic records (*7*) show the onset of warming by ~21 to 20 ka.

We suggest that the deglaciation and warming in NH and SH high-latitude regions by at least ~20 ka led to a reduction of Earth’s meridional thermal gradient (*40*) and that this reduced gradient influenced warming in the tropics (Fig. 4). The global atmosphere can be modeled as a “heat engine,” with net flow from warm “source” regions at low latitudes to cool heat “sinks” at high latitudes (*42*). The efficiency of heat flow is greatest when the temperature difference between source and sink regions is large and decreases when the temperature gradient between these regions is reduced. The export of heat from the tropics is dominated by the Hadley circulation, the strength of which is positively related to the meridional thermal gradient (*43*). This relationship is apparent in simulations of anthropogenic global warming that show decreased Hadley circulation strength as the thermal gradient between the tropics and the poles is reduced (*44*–*46*). By analogy, we infer that the insolation-induced warming of both polar regions marked by the recession of NH and SH ice sheets at ~21 to 20 ka influenced tropical warming through reducing the Hadley circulation strength and, thus, net tropical heat export. Slackened Hadley circulation also would have likely decreased poleward oceanic heat export by tropical surface currents (*47*, *48*), further reinforcing tropical warming. Tropical SSTs likewise began to rise at or just before ~19 ka (Fig. 4) (*7*).

One hypothesis arising from this mechanism is that tropical glacial recession should have occurred nearly coincident with, or immediately after, increases in high-latitude temperatures. Tropical glacial recession was underway at ~20 to 19 ka. In contrast, compilations of higher-latitude temperature records suggest high-latitude warming initiated at ~21 to 20 ka (Fig. 4). Future work testing this hypothesis should focus on further high-resolution reconstructions of tropical temperatures, including dating of tropical glacial extents in Africa and elsewhere in the global tropics, which will reduce uncertainties in the precise phasing of high- and low-latitude glacial fluctuations during and subsequent to the LGM. Nevertheless, our results show that tropical temperatures respond to remote, high-latitude climate forcing and that climate models of the last deglaciation should account for tropical warming before ~18 ka absent the influence of GHG forcing.

## MATERIALS AND METHODS

### Field methodology

We conducted three field seasons in the Mubuku, Bujuku, and Moulyambouli valleys of the Rwenzori Mountains (~0.3°N, 30°E) between 2012 and 2016. We sampled large (2 to 4 m in diameter) boulders on the crests of moraines that showed little or no sign of postdepositional movement or modification. We obtained ~0.5- to 1-kg pieces of the upper 3 to 5 cm of boulders using a hammer and chisel or a battery-powered hammer drill. We measured the surface dip of each sample using a handheld Suunto compass and any potential shielding by topography using a Suunto clinometer. Where possible, we sampled flat rock surfaces with no dip to minimize potential uncertainties in the shielding correction. We recorded the location and elevation of each sample at the time of collection using a handheld Garmin Global Positioning System (GPS). We averaged GPS measurements over the course of 2 to 5 min, sufficient to record ≥100 measurements. We then recorded the average location and elevation values for each sample (vertical error, ±3 m; horizontal error, ±1 m).

### Lab methodology

We measured the thickness of each sample to the nearest millimeter (±1 mm) using calipers before crushing and milling the sample to a grain size of 210 to 750 μm. We then put the 210- to750-μm grain-size fraction through a series of *o*-phosphoric acid, sodium hydroxide, and dilute hydrofluoric-nitric acid leaches to etch quartz and dissolve other minerals. Once we obtained pure quartz, we spiked the samples and a process blank with a known mass of ^9^Be carrier and digested them in hydrofluoric acid. We used a ^9^Be carrier developed from a deeply buried beryl crystal. For carrier concentrations, see table S1. We then used a modified version of the procedures described in (*49*) to isolate beryllium from each sample and the process blank. ^10^Be/^9^Be ratios of samples were measured at the Center for Accelerator Mass Spectrometry at Lawrence Livermore National Laboratory (LLNL) and normalized to the 07KNST3110 standard (table S1) (*50*). Once obtained, we subtracted the ^10^Be/^9^Be process blank ratio from the ^10^Be/^9^Be ratios of measured samples and used the blank-corrected sample ratios to determine the concentration of ^10^Be in each sample per gram of quartz. For process blank ratios, see table S1.

### ^10^Be age calculation

We calculated the Rwenzori ^10^Be ages using version 3 of the online exposure age calculator described by Balco *et al.* [(*51*) and subsequently updated] using a low-latitude, high-altitude ^10^Be production rate (*21*) and the time-independent St scaling scheme (tables S1 and S2 and fig. S1) (*23*–*25*). Two ^10^Be production rate calibrations from tropical high-altitude sites (both in South America) (*20*, *21*) yield the most robust calibration results when paired with St scaling, which suggests that this method may be most appropriate for sites such as the Rwenzori. We note that these ^10^Be production rate calibrations use glacial deposits that date to ~13 to 11 ka BP, younger than the LGM.

Assuming that changes in the geomagnetic field influenced the cosmic-ray flux and, thus, the ^10^Be production rate during and since the LGM, a time-dependent scaling scheme (e.g., “Lm” or “LSDn”) (*52*) may be more appropriate for calculating the Rwenzori ^10^Be ages. Time-dependent scaling schemes such as Lm and LSDn (*51*) yield less accurate calibrations of the Kelly *et al.* (*21*) dataset than St, with Lm scaling producing the greatest scatter in the resulting calibration data. Moreover, Lm scaling is based on paleomagnetic models (*51*); because the theorized impacts of changes in the magnetic field on nuclide production are greatest in the low-latitude regions (*53*), we suggest that Lm scaling is less appropriate than other scaling schemes for sites in the tropics. LSDn scaling yields more accurate calibration results than the Lm scaling and is based on the cosmic-ray flux rather than magnetic field models (*52*, *53*). We show the Rwenzori ^10^Be ages calculated using Lm and LSDn scaling in table S2. However, because of uncertainties in the ^10^Be production rate and scaling schemes in the low latitudes (*26*), we choose to base our interpretations on ^10^Be ages calculated using St scaling. Our interpretations regarding the timing of tropical glaciation (during the global LGM) and onset of deglaciation (at ~20 to 19 ka) do not change with LSDn-based calculations. Our interpretations would change using Lm scaling, but because of the relative inaccuracy of Lm when used to calibrate tropical exposure age data, we consider Lm less appropriate to use in this study.

We did not correct the Rwenzori ^10^Be ages for the potential influence of boulder surface erosion. Although many samples showed evidence of surface erosion (e.g., exfoliation, raised quartz veins), ^10^Be ages obtained from raised quartz veins are indistinguishable from those obtained from boulder surfaces. For example, on the Mahoma 4 moraine, samples RZ-16-49 (28,650 ± 570 years) and RZ-16-50 (30,100 ± 790 years) are from raised quartz veins (1 to 3 cm above the boulder surface) on gneissic boulders (table S2). The other four samples from the Mahoma 4 moraine are from gneissic boulder surfaces and yielded ages between ~29.9 and 27.5 ka. The mean age of the Mahoma 4 moraine including these quartz vein samples (28,960 ± 1020 years) is statistically indistinguishable from the mean age of the moraine with the quartz vein samples excluded (28,750 ± 1100 years).

In addition, we did not correct the Rwenzori ^10^Be ages for the influence of cover by snow and/or ice, soil, or vegetation. We never observed snow or ice cover at the sample sites and suggest that snow and ice do not persist on the ground for any length of time at the sample elevations (~2635 to 2990 m.a.s.l.) owing to the relatively warm temperatures and intense equatorial solar radiation. All samples were covered by soil, moss, and various amounts of other vegetation. However, on the basis of the consistency of ^10^Be ages on individual moraines, we believe that shielding by soil and vegetation was small or negligible.

The χ^2^ value of each moraine, except for the Mahoma 3 moraine, is lower than the expected value (table S3). This indicates that any age scatter in the ^10^Be ages on individual moraines can be explained by analytical uncertainty alone rather than by postdepositional processes or modification. We identified outliers in the dataset using Chauvenet’s criterion. After removing a single outlier (RZ-13-63) from the Mahoma 3 moraine ages, the χ^2^ value of the Mahoma 3 moraine is lower than the expected χ^2^ value. Sample RZ-13-63 from the Mahoma 3 moraine is the only sample identified as an outlier and is not included in our interpretations or discussion. All “mean ages” described in the text are arithmetic means of the sample ages discussed.

Certain samples were measured twice (i.e., two separate quartz aliquots from the same rock sample were processed and measured) to check for internal sample age consistency. Secondary aliquot measurements are marked as aliquot “a” in tables S1 and S2, and all ^10^Be ages are shown in tables S1 and S2. We used the secondary aliquot (aliquot “a”) measurements for our interpretations and discussion of ^10^Be ages from moraine Mahoma 0, because the primary sample measurements returned less scatter between ages. We do not base our larger interpretations on these samples, and we note that the aliquot “a” measurements for all other samples are within error of primary measurements.

### Recalculation of preexisting tropical ^10^Be datasets

We recalculated ^10^Be ages from prior studies on tropical glacier fluctuations using the same methodology described above for the Rwenzori ^10^Be ages. We provide a brief review of the sites and samples from prior studies within the Supplementary Materials of this manuscript. We used the St scaling scheme (*23*–*25*) for calculating the ^10^Be ages from prior work, but note that the differences in ^10^Be ages calculated using the Lm (*51*) and LSDn (*52*) scaling schemes are similar to those for the Rwenzori (see tables S1 and S4).

For the purpose of making the most direct comparisons possible in the discussion of the paper, we chose to include only prior work that used ^10^Be dating to develop glacial chronologies. Therefore, we did not include prior studies that used radiocarbon dating of organic material associated with moraines or surface exposure dating of moraines using other cosmogenic nuclides such as ^36^Cl and ^3^He. However, we note that these additional data neither alter nor counter our broader interpretations.

With the recalculated ^10^Be ages from prior studies in the tropics, we assigned moraine ages at sites using the following methodology. First, we excluded ^10^Be ages identified as outliers by the original authors. We also excluded ^10^Be ages that were not from moraines (i.e., ^10^Be ages of bedrock surfaces or boulders on bedrock surfaces). We then plotted the locations of ^10^Be ages in Google Earth and evaluated their glacial geologic contexts. We checked these locations using the original published maps of ^10^Be ages and glacial geomorphology to ensure that samples plotted correctly as reported in the prior studies. We assigned each sample to a moraine based on the original authors’ interpretations. However, where authors grouped ^10^Be ages of multiple landforms into a single mean age or moraine group, we attempted to assign ^10^Be ages to individual moraines using Google Earth. Once each ^10^Be age was assigned to a moraine, we calculated the mean age of the moraine. Descriptions of the recalculated ^10^Be datasets and assigned moraine ages are presented in the Supplementary Materials (see the “Tropical ^10^Be Site Descriptions” section). Because our study focuses on the LGM and the onset of the last deglaciation, we excluded ^10^Be ages >40 ka and mean moraine ages >30 ka, as well as ^10^Be ages <17 ka from prior studies.

Where possible for prior studies, we also assessed whether individual moraines mark the onset of recession from or from near the LGM maximum position of a given glacier. This was not always possible to determine because, at some sites, only lateral, composite moraines are dated. We detail these classifications in the Supplementary Materials. All recalculated ^10^Be ages and associated data are shown in table S4.

## Supplementary Material

http://advances.sciencemag.org/cgi/content/full/5/12/eaaw2610/DC1

Download PDF

Table S1

High-latitude warming initiated the onset of the last deglaciation in the tropics

## SUPPLEMENTARY MATERIALS

Supplementary material for this article is available at http://advances.sciencemag.org/cgi/content/full/5/12/eaaw2610/DC1 Supplementary Text Fig. S1. Camel plots showing probability-distribution curves for individual moraine ages with sample age statistics. Table S1. Sample information for Moulyambouli and Mahoma moraines. Table S2. Calculated ^10^Be ages from the Moulyambouli and Mahoma moraines. Table S3. Distribution of ^10^Be ages from the Moulyambouli and Mahoma moraines as presented in fig. S1. Table S4. Recalculated ^10^Be ages of tropical South American moraines. Table S5. Calibration dataset for Kelly *et al.* (*21*) as provided for use with version 3 of the online exposure age calculator described by Balco *et al.* (*51*) and subsequently updated. References (*55*–*66*)
