# Supplementary material for: High-latitude warming initiated the onset of the last deglaciation in the tropics
Source: Sci Adv. 2019 Dec 11;5(12):eaaw2610. doi: 10.1126/sciadv.aaw2610 (PMC6905867; doi:10.1126/sciadv.aaw2610)
Supplement: http://advances.sciencemag.org/cgi/content/full/5/12/eaaw2610/DC1 [file supp_5_12_eaaw2610__index.html]

Science Advances | Science AdvancesAAASSearchScience AdvancesMenu

## Supplementary Materials

**The PDFset includes:**

- Supplementary Text
- Fig. S1. Camel plots showing probability-distribution curves for individual moraine ages with sample age statistics.
- Legends for tables S1 to S5
- References (*55*–*66*)

Download PDF

**Other Supplementary Material for this manuscript includes the following:**

- Table S1 (Microsoft Excel format). Sample information for Moulyambouli and Mahoma moraines.
- Table S2 (Microsoft Excel format). Calculated 10Be ages from the Moulyambouli and Mahoma moraines.
- Table S3 (Microsoft Excel format). Distribution of 10Be ages from the Moulyambouli and Mahoma moraines as presented in fig. S1.
- Table S4 (Microsoft Excel format). Recalculated 10Be ages of tropical South American moraines.
- Table S5 (Microsoft Excel format). Calibration dataset for Kelly *et al.* (*21*) as provided for use with version 3 of the online exposure age calculator described by Balco *et al.* (*51*) and subsequently updated.

**Files in this Data Supplement:**

- Adobe PDF - aaw2610\_SM.pdf
