# Supplementary material for: High-latitude warming initiated the onset of the last deglaciation in the tropics
Source: Sci Adv. 2019 Dec 11;5(12):eaaw2610. doi: 10.1126/sciadv.aaw2610 (PMC6905867; doi:10.1126/sciadv.aaw2610)
Supplement: Download PDF [file aaw2610_SM.pdf]

Science Advances
AAAS

advances.sciencemag.org/cgi/content/full/5/12/eaaw2610/DC1

Supplementary Materials for

High-latitude warming initiated the onset of the last deglaciation in the tropics

Margaret S. Jackson*, Meredith A. Kelly, James M. Russell, Alice M. Doughty, Jennifer A. Howley, Jonathan W. Chipman, David Cavagnaro, Bob Nakileza, Susan R. H. Zimmerman

*Corresponding author. Email: margaret.s.jackson.gr@dartmouth.edu

Published 11 December 2019, *Sci. Adv.* **5**, eaaw2610 (2019)
DOI: 10.1126/sciadv.aaw2610

The PDF file includes:

Supplementary Text
Fig. S1. Camel plots showing probability-distribution curves for individual moraine ages with sample age statistics.
Legends for tables S1 to S5
References (*55*–*66*)

Other Supplementary Material for this manuscript includes the following:

(available at advances.sciencemag.org/cgi/content/full/5/12/eaaw2610/DC1)

Table S1 (Microsoft Excel format). Sample information for Moulyambouli and Mahoma moraines.
Table S2 (Microsoft Excel format). Calculated ^{10}Be ages from the Moulyambouli and Mahoma moraines.
Table S3 (Microsoft Excel format). Distribution of ^{10}Be ages from the Moulyambouli and Mahoma moraines as presented in fig. S1.
Table S4 (Microsoft Excel format). Recalculated ^{10}Be ages of tropical South American moraines.
Table S5 (Microsoft Excel format). Calibration dataset for Kelly *et al.* (*21*) as provided for use with version 3 of the online exposure age calculator described by Balco *et al.* (*51*) and subsequently updated.

Supplementary Text

Climatic Controls on Tropical Glaciation

Studies of modern (*12*, *61*) tropical glaciers show that temperature is a dominant influence on glacial mass balance in the humid inner tropics (10ºN-10ºS). In the Rwenzori, Taylor et al. (*61*) used remote sensing of recent glacial extent changes to argue that temperature is a primary control on glacial extent within the range. In South America, Sagredo and Lowell (*12*) analyzed recent glacial extent changes along the whole of the Andean Cordillera and showed that, in the inner tropics, glaciers are more sensitive to changes in temperature than to changes in precipitation. Rupper et al. (*62*) used mass balance modeling of Himalayan glacial systems to show the same temperature dominance on humid glacial systems.

Studies of past glacial extent changes suggest that temperature was a primary control on tropical glacial extent following the LGM. Jomelli et al. (*13*) analyzed the timing and extent of glacial fluctuations in the northern and southern South American tropics during the late-glacial (~15-11 ka) period. They showed that, although precipitation changes were regionally anti-phased between the northern and southern South American tropics (*13*, *63*), glaciers in both regions fluctuated similarly, suggesting that temperature was the dominant control on glaciers during this time.

Previous work in the Rwenzori showed that LGM glaciation took place contemporaneously with cold and dry conditions (*22*, and references therein). GDGT temperature records from tropical African lakes also indicate cold conditions in the region during the LGM (*33, 35, 64*). Records of precipitation from equatorial and subequatorial East Africa suggest that the region was relatively

dry during the LGM period with no indication of rapid precipitation changes coincident with the onset of deglaciation (*64, 65*). In tropical South America, precipitation patterns varied across the region during the LGM and at the onset of deglaciation (*63*), as did the onset of wetter conditions following the LGM. At Santiago Cave, Peru, and in the Salar de Uyuni, Bolivia, relatively dry conditions during the LGM ameliorated only after ~18 ka (*63*, and references therein), after the onset of glacial recession. In Venezuela, Cariaco Basin sedimentation rates indicate the region was relatively wet and gave way to drier conditions only after ~19 ka (*66*). The similarity in timing of the onset of deglaciation in the African and South American tropics suggests that glaciers across the low latitudes responded to a common driver. Because precipitation varied across the tropics during the LGM and at the onset of deglaciation, it is unlikely that precipitation was the primary forcing mechanism. By analogy with the above mentioned studies of modern and past tropical glacial mass balance sensitivities, we infer that past fluctuations of Rwenzori glaciers were influenced primarily by past tropospheric temperature changes.

Tropical ^{10}Be Site Descriptions

Here we describe the sites and samples from prior studies used within our analysis of tropical South American glacial extent changes. All "mean ages" described below are arithmetic means of the sample ages discussed.

Sierra Nevada, Venezuela (8ºN)

Wesnousky et al., 2012 [55]

Wesnousky et al. (2012) dated a series of moraines, terraces, and faulted features to assess the timing of regional glaciation and faulting. We recalculated the ^{10}Be ages from four moraines in

three separate catchments. In two catchments, ice retreated from its LGM maximum extent by ~19.0 ka. The third catchment may indicate recession after ~17.7 ka.

La Victoria Moraine (one moraine)

Four samples (VEN19, -20, -21, -23) date the La Victoria moraine and yield a mean age of ~19.0 ka. This suggests that deglaciation initiated at this site by ~19.0 ka. In GoogleEarth we observed two or three additional moraines distal to the La Victoria ridge. There are no ^{10}Be ages from these moraines.

Los Zerpas Moraines (two moraines)

Six samples (VEN25, -26, -27, -28; LZ09-01, -02) date the right- and left-lateral Los Zerpas moraines. For our analysis, we used one ^{10}Be age of the right-lateral moraine (VEN25, 20.5 ± 1.6 ka) and three ^{10}Be ages of the left-lateral moraine (VEN26-28, mean age ~19.2 ka). We excluded sample LZ09-02 (15.1 ± 1.1 ka) from the right-lateral moraine and sample LZ09-01 (16.8 ± 1.6 ka) from the left-lateral moraine because they are apparently young. These ^{10}Be ages were not identified as outliers by Wesnousky et al (2012). We also excluded sample VEN24 (47.0 ± 4.5 ka) from the left-lateral moraine. This ^{10}Be age was considered an outlier by Wesnousky et al. (2012). In GoogleEarth we did not observe additional moraines distal to the Los Zerpas moraines, although the heavily faulted nature of these moraines makes glacial geologic interpretations difficult. We consider the mean ~17.7 ka age of two samples on the left-lateral moraine more representative of the timing of post-LGM ice recession than the single ~20.5 ka sample age from the right-lateral moraine.

Faulted Moraine (one moraine)

One sample (VEN18) dates the faulted moraine and yields an age of ~21.7 ka. This ^{10}Be age is not discussed in the original paper. For our analysis, we used it as the mean moraine age. Whether this moraine represents the maximum LGM extent of ice in this catchment is unclear.

Sierra Nevada, Venezuela (8ºN)

Carcaillet et al. (2013) [56]

Carcaillet et al. (2013) dated two in a series of moraines that dam the modern Laguna de Mucubaji. Ice retreated from its LGM maximum extent by ~21 ka.

Laguna de Mucubaji (two moraines)

One sample (MU09-02) from the outermost dated moraine yields a ^{10}Be age of 22.6 ± 1.8 ka. One sample (MU09-01) from the innermost dated moraine yields a ^{10}Be age of 20.7 ± 0.7 ka. For our analysis, we used these ages as moraine ages. We consider the age of 20.7 ka as representative of recession from the LGM maximum extent.

Cajamarca, Peru (7ºN)

Shakun et al., 2015 [18]

Shakun et al. (2015) dated moraines in three former glacial catchments. In all catchments, ice retreated from its LGM maximum extent by ~22 ka.

North Camp Moraines (two moraines)

The North Camp moraines consist of paired right- and left-lateral moraines that mark the

apparent maximum extent of ice in the catchment during the LGM. Two ^{10}Be ages (MC-NC-5, -6) are from the right-lateral moraine and four ^{10}Be ages (MC-NC-7, -8, -9, -10) are from the left-lateral moraine. Shakun et al. (2015) excluded three ages (MC-NC-2, ~51.1 ka; MC-NC-3, ~68.4 ka; MC-NC-4, ~169.2 ka) because of presumed inherited ^{10}Be. For our analysis, we used these six ages that yield mean moraine ages of ~23.6 ka (right-lateral) and ~23.7 ka (left-lateral).

Galeno Moraines (three moraines)

The Galeno moraines are a series of ~5 nested moraines and are dated with nine ^{10}Be ages. One sample (MC-G-3; 18.9 ± 0.4 ka) is from the outermost dated terminal moraine. Two samples (MC-G-4, -2) are from a more proximal terminal moraine and yield a mean moraine age of ~17.3 ka. Five samples (MC-G-1, -6, -7, -8, -9) are from lateral moraines and yield a mean moraine age of ~20.4 ka. Shakun et al. (2015) excluded one age (MC-G-5, 46.0 ± 0.9 ka) because of presumed inherited ^{10}Be. For our analysis, we used the ^{10}Be age of MC-G-3 (18.9 ± 0.4 ka) as the mean moraine age. We averaged samples MC-G-4 and -2 for a mean moraine age of ~17.3 ka. In GoogleEarth we had difficulty observing the glacial geologic context of samples MC-G-1, -6, -7, -8, -9 and were not able to determine whether they are on a single or multiple moraines. Therefore, we assumed that they were on a single moraine and averaged their ^{10}Be ages to determine a mean moraine age of ~20.4 ka.

San Cirillo Moraines (not used)

The San Cirillo Moraines are within a catchment dotted by kettle lakes and hummocks. Eleven samples (SC-2 to -13) are from boulders on moraines and boulders perched on bedrock. These samples do not date a single landform. For our analysis, we excluded this dataset because we

could not assign a moraine age. However, in general these ^{10}Be ages indicate 1) that ice in the catchment achieved its LGM maximum extent prior to ~29 ka and 2) that ice retreated from its LGM maximum extent by ~22 ka.

Cordillera Blanca, Peru (9ºS)

Farber et al., 2005 [57]

Farber et al. (2005) dated moraines in two glacier catchments. In both catchments, ice was at or near its LGM maximum extent from at least ~29 to 21 ka, and recession from the LGM maximum extent was underway by ~21 ka.

Rurec Group 2, Quebrada Cojup (one moraine)

Five samples (HU-1, -2, -4, Peru-21, K-9) from a left-lateral moraine yield a mean moraine age of ~21.3 ka. For our analysis, we used samples (HU-1, -2, -4, and Peru-21) and considered sample K-9 (18.1 ± 1.0 ka) an outlier due to its apparently young age and its distance (~1 km) from the other four samples. The four samples (HU-1, -2, -4, and Peru-21) yield a mean moraine age of ~23.3 ka. We note that, when plotted in GoogleEarth, it is unclear whether these four samples are located on a single moraine or multiple moraines. For this reason, we do not use this moraine age as representative of the onset of recession from the LGM extent in our analysis.

Rurec Group 2, Quebrada Llaca (four moraines)

One sample (K-4) from the innermost dated right-lateral moraine yields an age of 22.7 ± 1.1 ka. We used this ^{10}Be age as the moraine age. One sample (K-3) from the next (more distal) right-lateral moraine yields an age of 24.5 ± 1.1 ka. We used this one ^{10}Be age as the moraine age. Two samples (K-5a, 29.4 ± 1.4 ka and K-5b, 29.0 ± 0.9 ka) from the outermost dated right-

lateral moraine yield a mean moraine age of 29.2 ± 0.3 ka.

Six samples (K-6a, -6b, -8a, -8b, -2, -7) are from the innermost dated left-lateral moraine and four of these samples (K-6a, -6b, -8a, -8b) were dated twice. For our analysis, we used all ten ^{10}Be ages to determine a mean moraine age of ~19.6 ka. We consider this age as representative of recession from the LGM maximum extent.

Cordillera Blanca, Peru (10ºS)

Smith & Rodbell, 2010 [58]

Smith & Rodbell (2010) dated a large left-lateral moraine in the Quenua Ragra Valley. Ice retreated from its LGM maximum extent by at least ~21 ka.

Quenua Ragra Valley (one moraine)

Four samples (JEU-33, -34, -35, -36) from a left-lateral moraine yield a mean moraine age of ~21.3 ka. For our analysis, we used the four ^{10}Be ages but note that, when plotted in GoogleEarth, the moraine appears to have multiple crests and it is unclear whether the samples are from a single crest or multiple crests.

Lake Junin, Peru (10ºS)

Smith et al., 2005a [16]

Smith et al. (2005a) dated moraines in four glacier catchments near Lake Junin. In all valleys, ice retreated from its LGM maximum extent between ~20 and ~17 ka.

Collpa Valley (one moraine)

Five samples (COL-01, -02, -03, -04, -07) from a left-lateral moraine yield a mean moraine age of ~26.8 ka.

Calcalcocha Valley (six moraines)

Seven samples (CAL-08 to -14) from the middle Calcolcocha Valley are from a series of low-relief terminal moraines and yield ^{10}Be ages between ~23.8 and 19.3 ka. Four of these samples (CAL-08, -11, -12, -13) from a terminal moraine yield a mean age of ~21.4 ka. In GoogleEarth the low-relief moraines are not clear and it is difficult to see where samples occur on the moraines. A single sample (CAL-14, 22.2 ± 0.8 ka) is from a more distal terminal moraine. We used this one ^{10}Be age as the mean moraine age. Two samples (CAL-09, -10) from a right-lateral moraine yield a mean moraine age of ~21.3 ka. We suggest that this age may be most representative of the timing of recession from the LGM maximum extent, as it is in stratigraphic order with the moraine described below (~19.5 ka). However, we do not use this age in our analysis.

Farther up the Calcolcocha Valley, seven samples date three nested moraines. One sample (CAL-07) is from the outermost moraine and yields a ^{10}Be age of 17.5 ± 1.6 ka. Three samples (CAL-04, -05, -06) from a moraine proximal to the ~17.5 ka moraine yield a mean age of ~17.4 ka. Three samples (CAL-01, -02, -03) from the innermost moraine yield a mean age of ~19.5 ka. Conservatively, we use this age (~19.5 ka) as the timing of recession from the LGM maximum extent.

There are additional dated moraines distal to the moraine in the middle Calcolcocha Valley. We did not include the ^{10}Be ages of these moraines in our analysis because they pre-date the LGM.

Antacocha Valley (eight moraines)

In the middle Antacocha Valley, eight samples (ANT-08 to -14) date a series of low-relief terminal moraines. We exclude these ages from our analysis because they predate the LGM.

In the upper Antacocha Valley, seven samples (ANT-01 to -07) date six moraines that mark the former terminal positions of a glacier in the valley. For our analysis, we used the seven ^{10}Be ages and assigned them to six moraines. Samples ANT-05 and -06 date the outermost moraine and yield a mean moraine age of ~18.3 ka. Sample ANT-04 (21.3 ± 0.9 ka) may also date this outermost ridge but, due to uncertainty in the glacial geomorphic context of the sample, we treat it as an individual mean moraine age. Sample ANT-03 (20.7 ± 0.6 ka) dates the next more proximal moraine. Roughly 100 m from ANT-03 on the same apparent moraine ridge, ANT-07 yields an age of 19.7 ± 0.7 ka. However, due to uncertainty in geomorphic context of the samples, we treat them as representative of individual moraine ridges. Sample ANT-02, which appears to be more proximal relative to samples ANT-03 and ANT-07, yields an age of 20.6 ± 0.6 ka. One sample (ANT-01) from the innermost dated moraine yields an age of 19.3 ± 0.7 ka. Conservatively, we treat the age of ~19.3 ka as representative of the timing of the timing of recession from the LGM maximum extent.

Alcacocha Valley (three moraines)

In the lower Alcacocha Valley, eleven samples (ALC-23 to -25, ALC-01 to -05, ALC-26 to -29) range in age from ~16.8 to ~34.1 ka. These samples are from former terminal moraines in the central portion of the valley. We exclude these ^{10}Be ages from our analysis because of the spread in ages and because these ages are out of stratigraphic order with other moraine ages in the valley.

In the middle Alcacocha Valley, two samples (AL006, 007) from a terminal moraine yield a mean moraine age of ~19.6 ka. Proximal to this moraine, one sample (AL010) from a separate moraine yields an age of 20.8 ± 0.5 ka. We consider the age of ~19.6 ka as most representative of the timing of recession.

Four samples (ALC-03 to -06) are from a moraine ~250 m away from the ~19.6 ka moraine and yield a mean moraine age of ~21.3 ka. This moraine may be a portion of the ~19.6 ka moraine, but due to uncertainty in the relationship between these moraine segments we treat them as separate landforms. We excluded sample ACL-07 (35.2 ± 1.0 ka) as an outlier.

In the upper Alcacocha Valley, seven samples (AL001-5, PE01-ALC-01, -2) yield ages between ~17.0 and 33.5 ka. We excluded these samples from our analysis because these ages are 1) out of stratigraphic order with other samples from the valley, 2) post-LGM, or 3) not associated with moraines when viewed in GoogleEarth.

Cordillera Oriental, Peru (14ºS)

Bromley et al., 2016 [19]

Bromley et al. (2016) dated three terminal moraines at Quebrada Tiratana and one right-lateral moraine near Laguna Aricoma. At both sites ice retreated from its LGM maximum extent by ~18.8 ka.

Quebrada Tirataña (three moraines)

Two samples (NT-11-05, -08) from the outermost dated moraine yield a mean age of ~27.0 ka. One sample (NT-11-13) from a more proximal moraine yields an age of 25.3 ± 0.3 ka. We used this one ^{10}Be age as the mean moraine age. One sample (NT-11-18) from the innermost dated

moraine yields an age of 21.6 ± 0.3 ka. We used this one ^{10}Be age as the mean moraine age.

Laguna Aricoma (one moraine)

Two samples (ARC-09-25, -26) from a right-lateral moraine yield a mean age of ~18.8 ka. This is the outermost moraine. There are numerous undated more proximal lateral moraines.

Milluni Valley, Bolivia (16ºS)

Smith et al., 2005b [17]

Smith et al. (2005b) dated two left-lateral moraines in the Milluni Valley that are ~200 m apart. Below we describe these moraines as the outermost and innermost moraines. In general, the ice was at or near its LGM maximum extent between ~30 and 18 ka.

Milluni valley (one moraine)

Seven samples (MIL-00-08 to -14) from the outermost moraine yield a mean age of 29.3 ± 2.5 ka. Seven samples (MIL-00-01 to -07) from innermost moraine yield a mean age of 30.5 ± 8.9 ka. Three ^{10}Be ages (MIL-00-01, 37.6 ± 1.0 ka; MIL-00-02, 35.6 ± 1.0 ka; MIL-00-04, 41.1 ± 1.5 ka) of the innermost moraine are older than the oldest age (MIL-00-13, 33.5 ± 0.9 ka) of the outermost moraine. Two ages from the innermost moraine (MIL-00-03D, 30.7 ± 0.8 ka and MIL-00-07, 31.7 ± 0.9 ka) are similar to those from the outermost moraine. For our analysis, we use the two ^{10}Be ages of the innermost moraine (MIL-00-05, 17.5 ± 0.5 ka; MIL-00-06, 19.6 ± 0.6 ka) and determine a mean moraine age of ~18.6 ka, which we take as the timing of recession from the LGM maximum extent at this site.

Cordillera Real and Cochabamba, Bolivia (15-17ºS)

Zech et al., 2007 [59]

Zech et al. (2007) dated moraines in Valle San Francisco and Valle Huara Loma, two separate catchments. Ice retreated from its LGM maximum extent by ~22 ka.

Valle San Francisco (three moraines)

Three samples (SF-41, -42, -43) from the outermost dated right-lateral moraine yield a mean age of ~34.6 ka. Two samples (SF-32, -33) from a more proximal right-lateral moraine yield a mean age of ~28.1 ka. Two samples (SF-12, -13) from a left-lateral moraine yield a mean age of ~27.4 ka. We do not consider these moraines to represent the onset of deglaciation from the LGM in this catchment.

Valle Huara Loma (one moraine)

Two samples (HH51, -52) from a right-lateral moraine segment yield a mean age of ~22.7 ka. Additional moraines at this site are dated by May et al., 2011 (described below).

Cordillera de Cochabamba, Bolivia (17ºS)

May et al., 2011 [60]

May et al. (2011) dated four moraines in Valle Huara Loma. Ice retreated from its LGM maximum extent between ~22 and ~20 ka.

Valle Huara Loma

One sample (HL11) from a left-lateral moraine yields an age of 28.8 ± 1.9 ka. Down valley from HL11, on a terminal moraine, sample HL12 yields an age of 20.3 ± 1.2 ka. We consider this age

the most representative of the timing of ice recession from the LGM maximum extent. Farther down valley, one sample (HL43) from a right-lateral moraine yields an age of 23.4 ± 1.1 ka. These moraines are proximal to the moraine dated by Zech et al. (2007) described above.

Two additional samples from a more distal right-lateral moraine (HL51, -52) yield a mean age of ~29.7 ka.

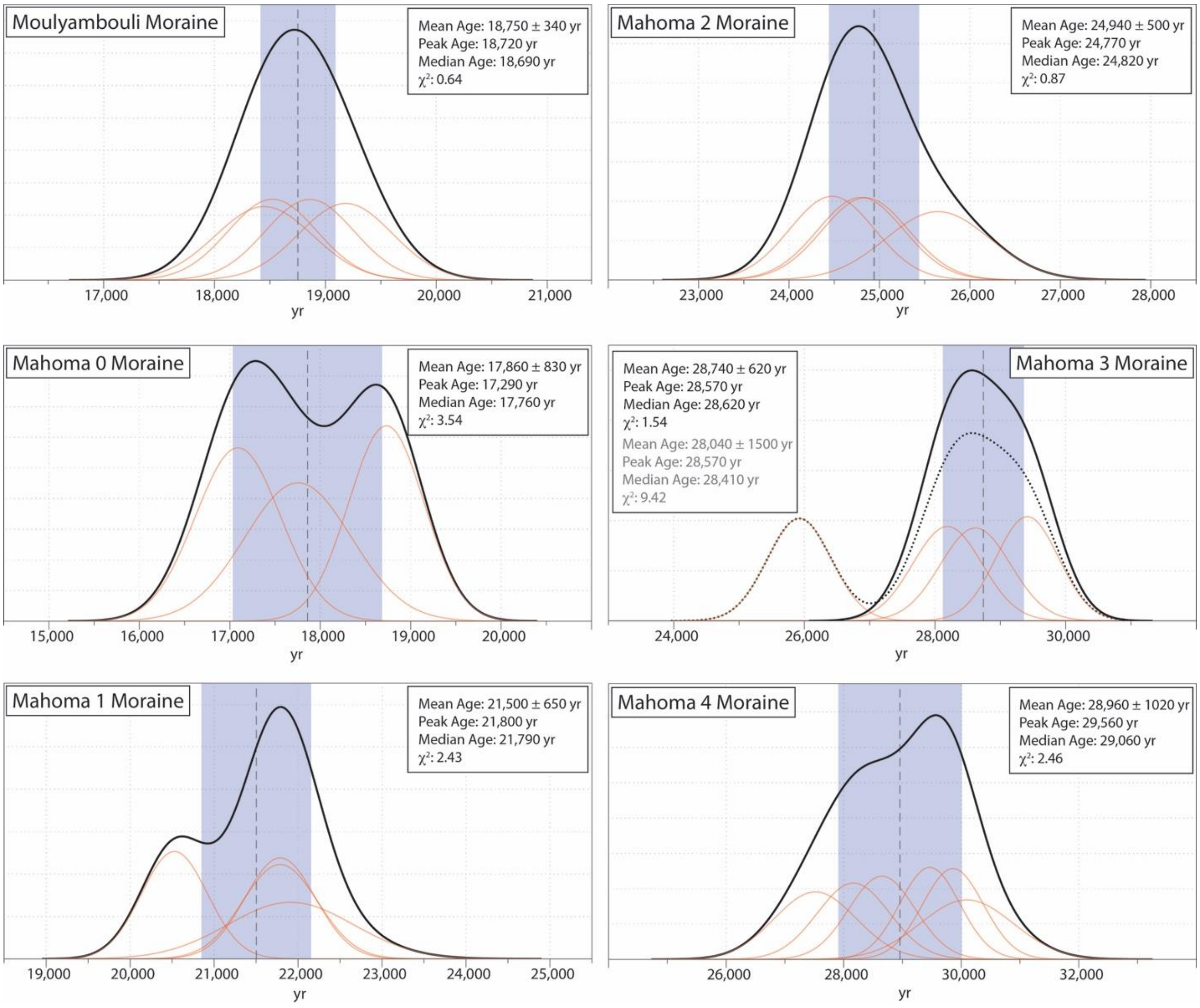

Fig. S1. Camel plots showing probability-distribution curves for individual moraine ages with sample age statistics. Time (yr) is on the x-axis and relative probability (unitless) is on the y-axis for each plot. Single sample ^{10}Be ages are represented by red lines, moraine ages by black lines. Dashed lines indicate the mean moraine age, bounded by 1-sigma standard deviation in blue. The dashed curve in the Mahoma 3 moraine plot indicates the age of the moraine prior to removal of outliers (statistics shown in gray).

Table S1. Sample information for Moulyambouli and Mahoma moraines. Table includes field measurement data, laboratory processing and preparation information, and beryllium ratio results from LLNL.

Table S2. Calculated ^{10}Be ages from the Moulyambouli and Mahoma moraines. Ages are reported using time-invariant ("St") and time-dependent ("Lm", "LSDn") scaling methods and a low-latitude, high-elevation production rate (*21*). Also shown are the internal and external calculated age errors for each scaling method. The mean age and standard deviation for each moraine are reported using "St" scaling. Aliquot 'a' samples are used for analysis and discussion of ages from the Mahoma 0 moraine, as primary sample aliquots (also reported here) returned abnormally low currents during measurement. All other aliquot 'a' samples are reported for other samples, but these are not included in moraine age analysis or discussed within the text.

Table S3. Distribution of ^{10}Be ages from the Moulyambouli and Mahoma moraines as presented in fig. S1. The ages shown here are calculated using "St" scaling.

Table S4. Recalculated ^{10}Be ages of tropical South American moraines. All ages are calculated using version 3 of the online exposure age calculator described by Balco et al. (*51*) and subsequently updated using a high-altitude, low-latitude production rate (*21*). All sample cells marked in green were included within this analysis. The table includes ages calculated using both "St", "Lm" and "LSDn" scaling methods. We report the mean age of each moraine using "St" scaling. Mean moraine ages in bold are the outermost identified moraine within a given catchment, if dated. Mean moraine ages in italics denote the outermost dated moraine within a catchment, though there are additional undated moraines outboard.

Table S5. Calibration dataset for Kelly *et al.* (*21*) as provided for use with version 3 of the online exposure age calculator described by Balco *et al.* (*51*) and subsequently updated. Formatted as presented for use online at calibration.ice-d.org.
